# Regiospecific formation of the nitromethyl-substituted 3-phenyl-4,5-dihydroisoxazole via [3 + 2] cycloaddition

**DOI:** 10.1007/s00706-018-2227-6

**Published:** 2018-08-20

**Authors:** Barbara Mirosław, Dmytro Babyuk, Agnieszka Łapczuk-Krygier, Agnieszka Kącka-Zych, Oleg M. Demchuk, Radomir Jasiński

**Affiliations:** 1 Department of Crystallography, Maria Curie-Sklodowska University, Maria Curie-Sklodowska St 3, 20-031 Lublin, Poland; 2 Institute of Biology, Chemistry and Bioresources, Chernivtsi National University, 2 Kotsyubynski Str., Chernivtsi, 58012 Ukraine; 3 Institute of Organic Chemistry and Technology, Cracow University of Technology, Warszawska St. 24, 31-155 Kraków, Poland; 4 Department of Organic Chemistry, Maria Curie-Sklodowska University, Gliniana St 33, 20-031 Lublin, Poland

**Keywords:** Isoxazole, Nitrocompounds, 3-Nitroprop-1-ene, DFT calculations, [3 + 2] Cycloaddition

## Abstract

**Abstract:**

5-(Nitromethyl)-3-phenyl-4,5-dihydroisoxazole was obtained as a product of a high-yielding [3 + 2] cycloaddition reaction of in situ-generated benzonitrile *N*-oxide and 3-nitroprop-1-ene. For the first time, the regiochemistry of this reaction was unambiguously proven by X-ray structural analysis. The quantum-chemical calculation performed at the M06-2X/6-31G(d) (PCM) theoretical level affords a basis for explaining the course of reaction as well as the nature of transition states. Next, further DFT calculations together with spectral data shed light on structural aspects of the product.

**Graphical abstract:**

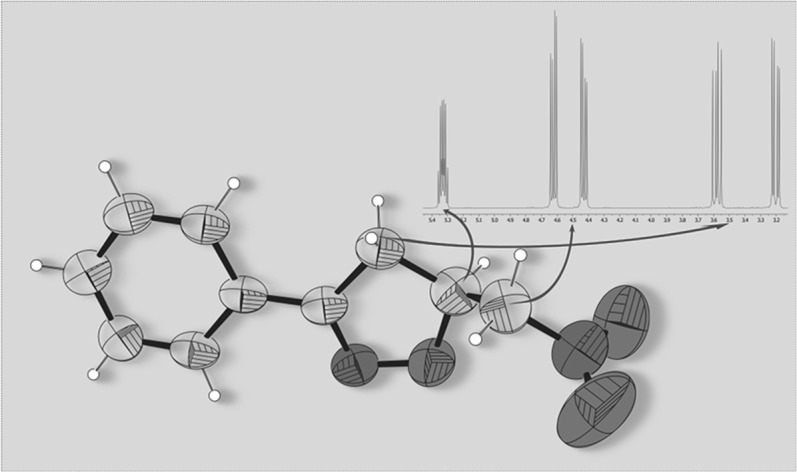

**Electronic supplementary material:**

The online version of this article (10.1007/s00706-018-2227-6) contains supplementary material, which is available to authorized users.

## Introduction

4,5-Dihydroisoxazoles (∆^2^-isoxazolines) are heterocycles with many important practical applications. They are used as parasiticidal agents in veterinary medicine [1] and β-adrenergic agonists [2]. Some of ∆^2^-isoxazolines exhibit inhibitory activity in tubulin polymerization [3] and anticancer activity against human prostate cancer cell lines [4]. The liquid crystalline properties of ∆^2^-isoxazolines are also known [5]. The synthetic potential of the use of ∆^2^-isoxazolines was explored in syntheses of *β*-hydroxyketones, *γ*-aminoalcohols, *α*,*β*-unsaturated oximes, and *β*-hydroxynitriles [6]. Applications of nitrosubstituted isoxazole derivatives in the field the chemistry and biochemistry are additionally also a logical consequence of (a) the wide range of theoretically possible channels of transformation of nitro group into other functional groups [7, 8] and (b) the influence of the nitro group on the biological activity [9, 10].

There are many efficient methods for preparation of nitrosubstituted 4,5-dihydroisoxazoles; the most universal one is [3 + 2] cycloaddition (32CA) between nitrile *N*-oxides as three atom components (TACs) [11, 12] and conjugated nitroalkenes (CNA) [13–15]. At the same time, nitromethyl-substituted 4,5-dihydroisoxazole is very poorly studied. Only one incidental synthesis of a compound from this group (nitromethyl-3-phenyl-4,5-dihydroisoxazole) has been briefly reported [16] to date. Unfortunately, the regiochemistry of that reaction was not reliably supported by structural analysis of the product formed: only low-resolution ^1^H and partial description of IR spectra have been presented. The proposed structure of the product was based rather on intuitive considerations than experimental data analysis.

Herein, we present the results of structural analysis of nitromethyl-3-phenyl-4,5-dihydroisoxazole, which was obtained in a 32CA reaction between in situ-generated benzonitrile *N*-oxide (**2**) and 3-nitroprop-1-ene (**3**) (Scheme 1).

**Figure:**
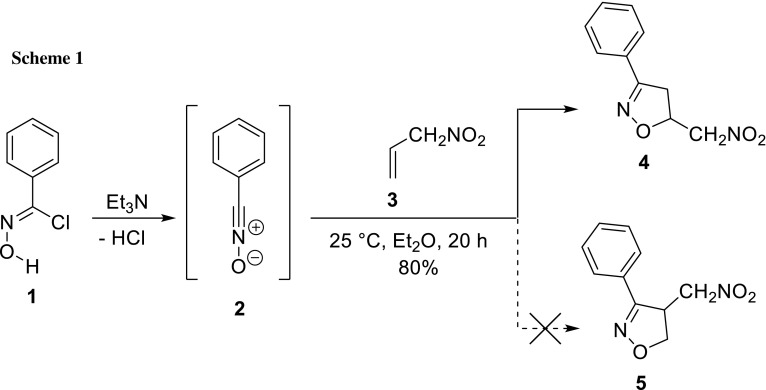

The results obtained provide a valuable background for understanding of the chemistry of nitromethyl-4,5-dihydroisoxazoles; they are also useful for better understanding of other 32CA processes involving nitroallylic systems, which are still only rarely used in cycloaddition reactions [17].

## Results and discussion

In the first stage, we prepared 3-nitroprop-1-ene (**3**) in a reaction between 3-bromoprop-1-ene and silver nitrite. The second component for the 32CA reaction, benzonitrile *N*-oxide (**2**), was generated in situ from phenylhydraxamoyl chloride. Next, we performed cycloaddition between **2** and **3**. The reaction was monitored by HPLC. It was found that the reaction was completed after 20 h. HPLC analysis of the crude reaction product confirmed the presence of unreacted residual nitroalkene and a single adduct formed. The product was isolated with sufficient purity for a full set of structural analyses using LC–MS IT-TOF, IR, and NMR techniques (please see for experimental section and Supplementary Materials for details).

The recorded NMR spectra of nitromethyl-substituted 3-phenyl-4,5-dihydroisoxazole (please see for experimental section and Supplementary Materials for details) could be compared with spectra of it known analogues for which both C5 and C4 substituted isomers were reported. In contrast to the substituted at C5 position isomers in the literature there are only a few relevant examples of C4 substituted compounds, which NMR spectra were reported. The spin system read from the ^1^H NMR spectrum of studied nitromethyl-substituted 3-phenyl-4,5-dihydroisoxazole corresponds some better to that, reported for 5-(hydroxymethyl)-3-phenyl-4,5-dihydroisoxazole [18], then for 4-(hydroxymethyl)-3-phenyl-4,5-dihydroisoxazole [19], but the signals corresponded to the both CH_2_ and H5 protons of nitromethyl-substituted compounds are shifted into the low field significantly compare to the hydroxymethyl-substituted analogues. Surprisingly, in the ^13^C NMR spectrum of 5-(hydroxymethyl)-3-phenyl-4,5-dihydroisoxazole, chemical shift of C5 carbon is situated in significantly lower field then corresponding signal of nitromethyl-substituted compound (81.2 vs. 76.9 ppm), in the same tome ^13^C NMR spectrum of C4 substituted isomer had not been reported. The comparison of ^1^H and ^13^C NMR of 5- [20] and 4- [21] hydroxymethyl-substituted 3-(*p*-methoxyphenyl)-4,5-dihydroisoxazoles was even less informative due to the signals overlapping and complicated structure of multiplets they formed. Any other pair of isomeric C4 and C5 substituted analogues with known spectral data were found in the literature.

The mentioned difficulties in comparison of the NMR spectra left some doubts that the collected data are sufficient to elucidate the structure of nitromethyl-substituted 3-phenyl-4,5-dihydroisoxazole surely. Therefore, to resolve this issue, we performed X-ray diffraction structural analysis.

**Fig. 1:**
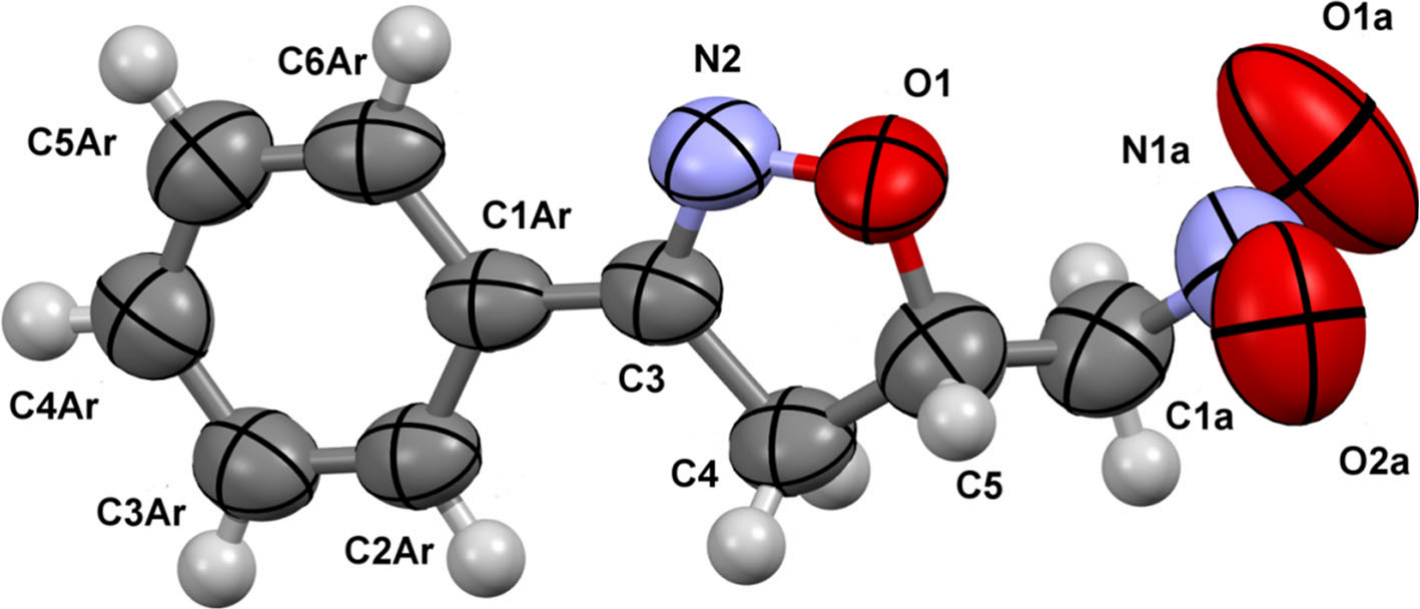
Molecular structure of **4**

In the X-ray diffraction experiment, we have confirmed unambiguously the molecular structure of 3-phenyl-5-(nitromethyl)-4,5-dihydroisoxazole corresponding to the structure of **4** in Scheme 1 (Fig. 1, Table 1). Although the geometry of isoxazole ring shows high diversity in bond length and valence angle values depending on the kind and the localization of substituents, the data for isoxazole ring in molecule **4** are within the ranges of values found in 751 structures deposited in the Cambridge Structural Database (CSD ver. 5.39 with update Feb. 2018) [22] (see Supplementary Materials). The isoxazole and phenyl rings in **4** are nearly coplanar (twisted by 5.2(2)°). The mean plane of the nitromethyl group (defined through atoms C1A, N1A, O1A, and O2A) is rotated by 63.7(5)° in relation with the isoxazole ring. In the CSD, there are no examples of nitromethyl-4,5-dihydroisoxazoles, but to show the freedom of rotation for the nitromethyl substituent in regard to the single bond (C5–C1) linking these two molecular fragments, we have made a search for compounds having 5-membered ring substituted by nitromethyl group. The values found for torsions between 5-membered ring and the nitromethyl group (–CNO_2_) were in the ranges of 12°–90° for 73 found crystal structures.

**Table 1:** Selected bond lengths of molecule **4**

Bond	Length/Å	Bond	Length/Å
O1–N2	1.380(6)	C5Ar–C6Ar	1.366(8)
O1–C5	1.452(7)	C3Ar–C4Ar	1.376(8)
N2–C3	1.274(6)	C4Ar–C5Ar	1.353(8)
C3–ClAr	1.446(8)	C5–C4	1.532(8)
C3–C4	1.508(7)	C1a–C5	1.434(8)
C1Ar–C2Ar	1.425(7)	C1a–N1a	1.504(10)
C1Ar–C6Ar	1.433(8)	N1a–O2a	1.186(9)
C2Ar–C3Ar	1.358(7)	N1a–O1a	1.117(9)

The conjugation of both rings results in shortening of the linkage bond C3–C1Ar (1.45(1) Å) and lengthening of the C1Ar–C2Ar and C1Ar–C6Ar bonds in the aromatic ring of up to 0.08 Å in regard to the shortest bond length within this ring. The HOMA index calculated for the benzene ring is only 0.74, showing a high degree of dearomatization and confirming the changes in the electronic structure of the molecule [24].

**Fig. 2:**
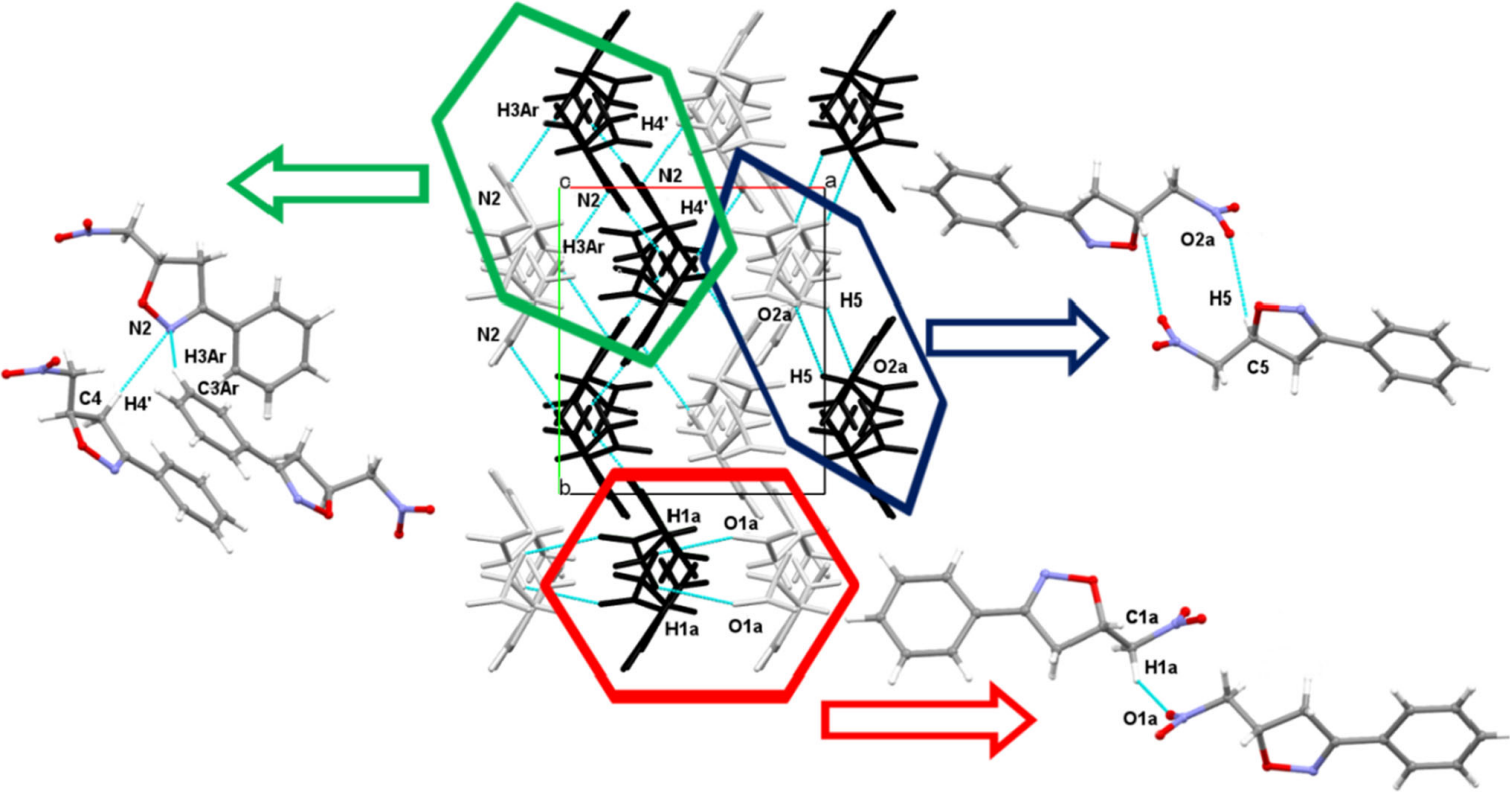
Crystal packing (view along the *c*-axis) and selected intermolecular interactions in **4**. Two directions of columns of molecules oriented in a head-to-tail manner are marked in grey and black

In the crystal, the molecules lie parallel along the *c-*axis in a “head-to-tail” manner (Fig. 2). They form *zig*-*zag* layers of molecules oriented in the same direction interacting only through a weak C4–H4′…N2 hydrogen bond within the layer (Table 2). In the neighbour layers, the molecules are facing in the opposite direction. Between the layers, the molecules contact through one cyclic C5–H5…O2a and two linear C3Ar–H3Ar…N2 and C1a–H1a…O1a hydrogen bonds. Although the molecule has a planar structure, there are no *π*…*π* interactions. This crystal arrangement favours the twin formation.

**Table 2:** Selected geometric parameters of intermolecular contacts in crystal **4**

D–H…A^#^	*d*(H…A)/Å	*d*(D…A)/Å	∠DHA/°	Symmetry code#
C5–H5…O2a^#^	2.71	3.424(2)	130	− *x*, − *y*, − *z*
C3Ar–H3Ar…N2^#^	2.72	3.504(2)	143	− *x*, *y* + 1/2, − * z*+ 1/2
C4–H4′…N12^#^	2.67	3.626(2)	169	− *x* + 1/2 + 1, + *y*+ 1/2, +*z*
C1a–H1a…O1a^#^	2.73	3.437(2)	130	*x* − 1/2, − *y *+ 1/2, − *z*

Next, we analyzed the mechanistic aspects of both theoretically possible competitive channels of the reaction between 3-nitroprop-1-ene and benzonitrile *N*-oxide. For this purpose, the results from DFT calculations were used (Tables 3, 4).

**Table 3:** Energetic parameters for [3 + 2] cycloadditions leading to 5-(nitromethyl)-3-phenyl-4,5-dihydroisoxazole (**4**) and regioisomeric 4-(nitromethyl)-3-phenyl-4,5-dihydroisoxazole (**5**) in diethyl ether according to M06-2X/6-31G(d) (PCM) calculations (respective parameters for reactions in more polar solvents are available in the Supplementary Materials)

Transition	∆*H*/kJ mol^−1^	∆*G*/kJ mol^−1^	∆*S*/J mol^−1^ K^−1^
2 + 3 → MC	− 27.2	19.3	− 156.2
MC → TSA	76.2	87.1	− 192.8
2 + 3 → 4	− 213.0	− 151.0	− 208.0
MC → TSB	84.6	96.1	− 194.7
2 + 3 → 5	− 201.3	− 139.0	− 208.6

**Table 4:** Most important parameters for the key structures of [3 + 2] cycloadditions leading to 5-(nitromethyl)-3-phenyl-4,5-dihydroisoxazole (**4**) and regioisomeric 4-(nitromethyl)-3-phenyl-4,5-dihydroisoxazole (**5**) according to M06-2X/6-31G(d) (PCM) calculations (respective parameters for reactions in more polar solvents are available in the Supplementary Materials)

Structure	C3–C4		C5–O1		GEDT/e	Imaginary frequencies/cm^−1^
	*r*/Å	l^a^	*r*/Å	l^a^		
TSA	2.192	0.549	2.281	0.416	0.002	− 462.63
**4**	1.511		1.440			
TSB	2.213	0.540	2.221	0.457	0.005	− 475.79
**5**	1.516		1.439			

^a^$l_{X - Y} = 1 - \frac{{r_{X - Y}^{TS} - r_{X - Y}^{P} }}{{r_{X - Y}^{P} }}$, where $r_{X - Y}^{TS}$ is the distance between the reaction centers *X* and *Y* in the transition structure and $r_{X - Y}^{P}$ is the same distance in the corresponding product

It was found that (regardless of the polarity of solvent) both reactions proceeded according to a one-step mechanism. In particular, only one transition state (TS) was localized between the valleys of individual substrates and products. However, the first reaction stage is always the formation of a pre-reaction complex (MC). This is accompanied by reduction of the enthalpy of the reaction system by about a 27 kJ/mol. In the MC, new σ-bonds are not formed. Distances between reaction centers exist beyond the area which is typical for new bonds in transitions states.

Further transformation of the reaction system proceeds into the transition state. In a weakly polar ethereal solution, this is accompanied by an increase in the enthalpy of the reaction system by 87.1 kJ/mol on path A and by 96.1 kJ/mol on path B (Table 3). In a strongly polar aqueous solution, these parameters of activation are equal to 85.6 and 97.0 kJ/mol, respectively. In general, polar solvents increase slightly the activation energy and decrease the exothermic character of 32CA reactions as a consequence of a better solvation of the TACs than the low polar TSs and cycloadducts [25]. However, irrespective of the polarity of solvents, the reaction channel leading to a 4-nitromethyl adduct should be considered as evidently unfavoured from the kinetic point of view. This conclusion correlates well with the results from the experimental study, which shows clearly that 5-nitromethyladducts is the only reaction product.

**Fig. 3:**
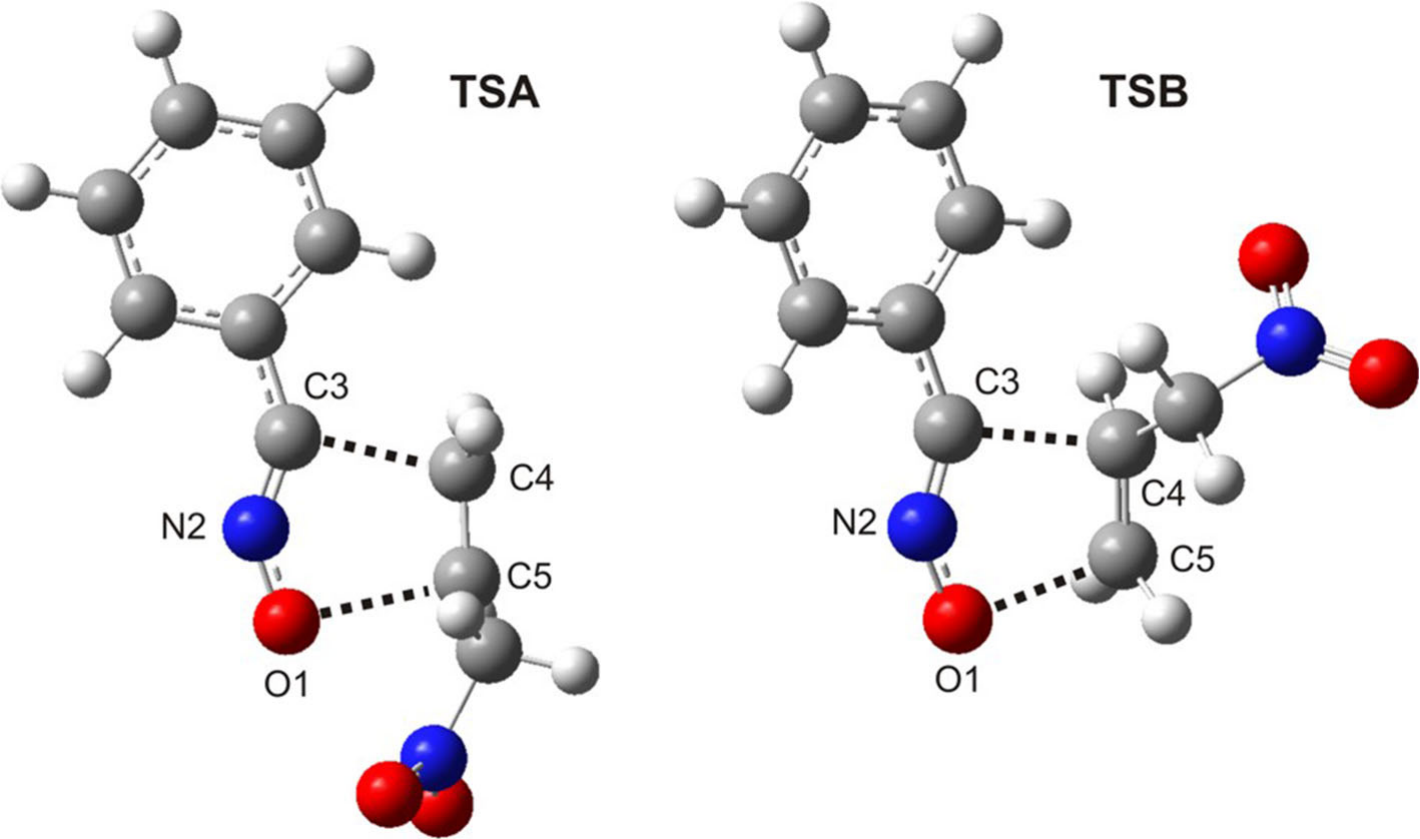
Views of transition states of cycloaddition between 3-nitroprop-1-ene and benzonitrile *N*-oxide in a diethyl ether solution according to M06-2X/6-31G(d) (PCM) calculations

Structurally, all transition states are very similar. In TSs, two new single bonds are formed (Fig. 3). These are bonds C3–C4 and C5–O1. Their degree of development is very similar, contrary to analogous transition states in cycloaddition reactions involving conjugated nitroalkenes [26–30]. Subsequently, the low values of GEDT [31] indices clearly confirm the non-polar nature of all considered reactions, even in the aqueous solution (see Supplementary Materials). In general, polar solvents increase slightly the activation energy and decrease the exothermic character of 32CA reactions as a consequence of a better solvation of the TACs than the low polar TSs and cycloadducts (see [28]).This conclusion is full agreement with fundamental rules of molecular electron density theory. In particular, Domingo has recently established a powerful classified of the [3 + 2] cycloaddition (32CA) reactions in base the structure of the TACs [32]. In this approach, nitrile *N*-oxides are classified as zwitterionic TACs, participating in zw-type 32CA reactions [33, 34]. This type of 32CA reaction demands the nucleophilic activation of the TAC and the electrophilic activation of the ethylene, or vice versa, to favour the 32CA reaction through a polar process [35]. Analysis of the conceptual DFT (CDFT, [36]) indices at the ground state of the reagents allows anticipate the reactivity of the species involved in a zw-type 32CA reaction. Thus, the low electrophilic (*ω* = 1.46 eV [26]) and nucleophilic character (*N* = 2.78 eV [26]) of benzonitrile *N*-oxide disable its participation in polar process, in spite of the high electrophilic character of 3-nitroprop-1-ene (*ω* = 1.92 [17]). These behaviours make the non-participation of nitrile *N*-oxides in polar zw-type 32CA reactions.

It should also be noted at this point that the optimized structure of 5-(nitromethyl)-3-phenyl-4,5-dihydroisoxazole (**4**) is close to that obtained from the X-ray diffraction structural analysis.

**Fig. 4:**
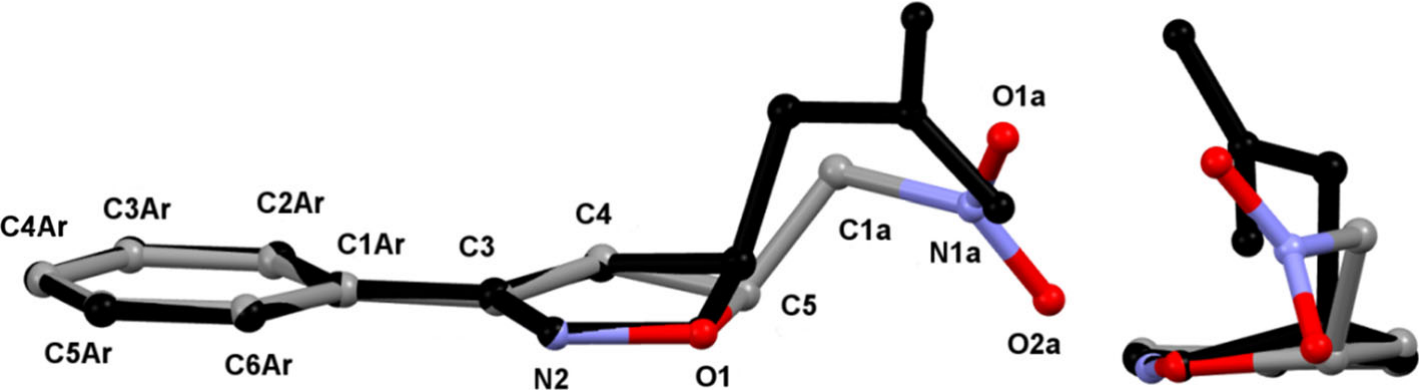
Two views of the best fitting of the experimental (colour) and theoretical (black, data for the diethyl ether environment) molecular structures

The molecular overlay of the experimental and calculated structures shows that the main geometric difference lies in the orientation of the nitromethyl substituent (Fig. 4). Both the rings are nearly coplanar. The isoxazoline ring is less planar in the calculated structures than in the crystal. The root mean square deviations (RMSD) for the bond lengths between the experimental and calculated geometry were 0.0915 Å for all solvents. The RMSD values were 2.198°, 2.181°, 2.172°, 2.170°, and 2.177° for valence angles and 11. 33°, 11.42°, 11.46°, 11.49°, and 11.50° for dihedral angles for diethyl ether, dichloromethane, acetone, nitromethane, and water, respectively. This geometric discrepancy may be regarded as a consequence of the existence of a crystal field along with the intermolecular interactions, which influence the molecular geometry.

## Conclusion

Our comprehensive structural analysis shed light on the regiochemistry of formation of 5-(nitromethyl)-3-phenyl-4,5-dihydroisoxazole in the [3 + 2] cycloaddition between benzonitrile *N*-oxide and 3-nitroprop-1-ene. It was found that the structure of 5-(nitromethyl)-3-phenyl-4,5-dihydroisoxazole should be assigned to the product. This conclusion is in full agreement with the results from DFT calculations, which also suggest relatively higher stability of the 5-nitromethyl isomer in the relation to the 4-nitromethyl one. It should be underlined that, for the first time, we report the crystal structure of a nitromethyl-substituted isoxazole compound. The rod-like shape of the molecule in combination with the layer type of the crystal packing implies the twin formation. Noteworthy, the conjugation of the aromatic and isoxazole rings results in dearomatization of the benzene ring and in some changes in the geometry and bond orders of the heterocyclic ring and the linking single bond.

The results obtained provide a valuable background for understanding of the chemistry of nitromethyl-4,5-dihydroisoxazoles. They are also useful for better understanding of other 32CA processes involving nitroallylic systems.

## Experimental

Melting points were determined on a Boetius apparatus. Elemental analysis was performed on a Perkin-Elmer PE-2400 CHN apparatus. The IR spectrum was recorded on an FTS Nicolet IS 10 apparatus. ^1^H NMR (500 MHz) and ^13^C NMR (125 MHz) spectra were taken on a Bruker Avance 500 MHz spectrometer calibrated to residual solvent peaks at 7.27 and 77.00 ppm for ^1^H and ^13^C in CDCl_3_. The chemical shifts are reported in ppm, and the following abbreviations are used in reporting the NMR data: d (doublet), dd (doublet of doublets), and m (multiplet). Coupling constants (*J*) are given in Hz. Spectra are reported as follows: chemical shift (*δ*, ppm), multiplicity, integration, and coupling constants (Hz). Liquid chromatography (HPLC) was done using a Knauer apparatus equipped with a UV–Vis detector. For monitoring of the reaction progress and postreaction mixtures, a LiChrospher 18-RP 10 μm column (4 × 240 mm) and 70% methanol as the eluent at a flow rate of 1.3 cm^3^ min^−1^ were used. MS spectra were recorded on a Shimadzu LCMS IT-TOF spectrometer equipped with a Kinetex^®^ 2.6 µm C18 100 Å, LC column 100 × 2.1 mm, eluted with 50% aqueous acetonitrile at a flow rate of 0.3 cm^3^ min^−1.^

### 3-Nitroprop-1-ene (3)

The powdered silver nitrite (50 g) was suspended in 100 cm^3^ of anhydrous diethyl ether at − 5 °C. Next, 35 g of 3-bromoprop-1-ene was slowly added dropwise, while the temperature was maintained at − 5 °C. The mixture was then left at 0–5 °C for 24 h, and next for 48 h at room temperature. The reaction mixture was filtered and the residue was washed with the diethyl ether. The filtrate and the ethereal extract were combined and the solvent was evaporated on a rotary evaporator. The liquid residue was distilled under reduced pressure. 7.2 g (29%) of 3-nitroprop-1-ene were obtained as a yellow liquid with a characteristic odor (bp. 35 °C/10 Torr; Ref.: 44 °C/27 Torr [37]).

### 5-(Nitromethyl)-3-phenyl-4,5-dihydroisoxazole (4, C_10_H_10_N_2_O_3_)

A mixture of 40 mmol of 3-nitroprop-1-ene and 20 mmol of phenylhydraxamoyl chloride (which was obtained via a typical procedure from benzaldoxime [38]) in 100 cm^3^ of dry Et_2_O was stirred and cooled down to 0 °C. 20 mmol of Et_3_N in 10 cm^3^ of Et_2_O was added dropwise and the mixture was stirred at r.t. for 20 h. The organic layer was separated from the postreaction mixture, dissolved in DCM, washed with water, and dried over MgSO_4_. The solvent was evaporated to dryness and residues were crystallized from EtOH. Yield 80%; white crystals; *m.p.*: 94–95 °C; ^1^H NMR (500 MHz, CDCl_3_): *δ* = 7.59 (m, 2H, Ar), 7.37 (m, 3H, Ar), 5.33 (dddd, 2H, *J *=10.6, 6.6, 6.3, 6.2 Hz, H5), 4.62 (dd, 1H, *J* = 13.2, 6.6 Hz, H6a), 4.43 (dd, 1H, *J* = 13.2, 6.3 Hz, H6b), 3.58 (dd, *J* = 17.0, 10.6 Hz, 1H, H4a), 3.20 (dd, *J* = 17.0, 6.2 Hz, 1H, H4b) ppm; ^13^C NMR (125 MHz, CDCl_3_): *δ* = 156.9 (C3), 130.8 (Ar_C4′_), 128.9 (Ar_C3′+C5′_), 128.4 (Ar_C1′_), 126.9 (Ar_C2′+C6′_), 76.8(C5) 76.1 (–CH_2_–), 38.7 (C4) ppm; IR (KBr): $\bar{\nu }$ = 3438, 2927, 2359, 1601, 1545, 1497, 1446, 1412, 1387, 1360, 1224, 1177, 1077, 1012, 912, 893, 822, 769, 712, 692, 546, 483, 438 cm^−1^; HRMS (ESI): *m/z* = 207.0758, calc. for [C_10_H_10_N_2_O_3_ + H]^+^ 207.0764, diff. = 2.90 ppm.

### X-ray crystallography

Crystals of **4** were obtained by recrystallization from ethanol. The crystals were twinned; however, the diffraction data produced good quality electron density maps that allowed us to unambiguously determine the structural model of the crystal (Table 5). The diffraction data were collected at room temperature on an XtaLAB AFC11 (RCD3): quarter-chi single diffractometer using CuK*α* radiation (*λ *= 1.54184 Å). Using Olex2 [37], the structure was solved with the ShelXT [39] structure solution program using intrinsic phasing and refined with the Olex2.refine refinement package using Gauss–Newton minimisation.

**Table 5:** Crystal data and structure refinement parameters for 5-(nitromethyl)-3-phenyl-4,5-dihydroisoxazole (**4**)

Formula	C_10_H_10_N_2_O_3_
Formula weight	206.20
Temperature/K	295
Crystal system	Orthorhombic
Space group	*Pbca*
*a*/Å	8.5754(11)
*b*/Å	9.8973(9)
*c*/Å	23.684(3)
*V*/Å^3^	2010.2(4)
*Z*	8
*ρ*_calc_/g cm^−3^	1.3626
*μ*/mm^−1^	0.861
*F*(000)	867.1
Crystal size/mm^3^	0.01 × 0.2 × 0.2
Radiation	Cu K*α* (*λ* = 1.54184)
2*θ* range for data collection/°	7.46 to 145.58
Reflections collected	28,948
Independent reflections	1853 [*R*_int_ = 0.3519, *R*_sigma_ = 0.0552]
Data/parameters	1853/135
Goodness-of-fit on *F*^2^	0.817
Final *R* indexes [*I *≥ 2*σ* (*I*)]	*R*_1_ = 0.0935, *wR*_2_ = 0.2952
Largest diff. peak/hole/e Å^−3^	0.36/− 0.24
CCDC no.	1576601

CCDC 1576601 contains supplementary crystallographic data for this paper. These data can be obtained free of charge via http://www.ccdc.cam.ac.uk/conts/retrieving.html (or from the Cambridge Crystallographic Data Center, 12, Union Road, Cambridge CB2 1EZ, UK; fax: +44 1223 336033).

### DFT calculations

The quantum-chemical calculations reported in this paper were performed on “Zeus” and “Prometheus” supercomputers in the “Cyfronet” regional computer center in Cracow. The M06-2X [40] and B3LYP functionals with 6-31G(d) basis set implemented in the GAUSSIAN 09 package [41] was used. It should be underlined that M06-2X functional is dedicated for precise energetic considerations [40, 42] and has recently been [26, 43–45] applied for analysis of several structural and energetic properties of compounds with similar structural moieties.

## Electronic supplementary material

Below is the link to the electronic supplementary material.

Supplementary material 1 (DOCX 3330 kb)
