# Supplementary material for: Regiospecific formation of the nitromethyl-substituted 3-phenyl-4,5-dihydroisoxazole via [3 + 2] cycloaddition
Source: Monatsh Chem. 2018 Aug 20;149(10):1877–84. doi: 10.1007/s00706-018-2227-6 (PMC6133101; doi:10.1007/s00706-018-2227-6)
Supplement: Supplementary file 1 — Supplementary material 1 (DOCX 3330 kb) [file 706_2018_2227_MOESM1_ESM.docx]

SUPPLEMENTARY MATERIALS

Regiospecific formation of the nitromethyl-substituted

3-phenyl-4,5-dihydroisoxazole via [3+2] cycloaddition

**Barbara Mirosław^1^ ● Dmytro Babyuk^2^ ● Agnieszka Łapczuk-Krygier^3^ ● Agnieszka Kącka-Zych^3^ ● Oleg M. Demchuk^4^ ● Radomir Jasiński^3^**

🖂 Radomir Jasiński

radomir@chemia.pk.edu.pl

^1^ Department of Crystallography, Maria Curie-Sklodowska University,

Maria Curie-Sklodowska St 3, 20-031 Lublin, Poland

^2^ Institute of Biology, Chemistry and Bioresources, Chernivtsi National University, 2 Kotsyubynski Str., Chernivtsi 58012, Ukraine

^3^ Institute of Organic Chemistry and Technology, Cracow University of Technology, Warszawska St. 24, 31-155 Cracow, Poland

^4^ Department of Organic Chemistry, Maria Curie-Sklodowska University, Gliniana St 33, 20-031 Lublin, Poland

**[1] Synthesis and properties of**

3-phenyl-5-nitromethyl-4,5-dihydroisoxazole

Analytic techniques

Melting points were determined on a Boetius apparatus and were uncorrected. Elemental analysis was performed on a Perkin-Elmer PE-2400 CHN apparatus. The IR spectrum was recorded on a FTS Nicolet IS 10 apparatus. ^1^H NMR (500 MHz) and ^13^C NMR (125 MHz) spectra were taken on a Bruker Avance 500 MHz spectrometer calibrated to residual solvent peaks at 7.27 ppm and 77.00 ppm for ^1^H and ^13^C in CDCl_3_. The chemical shifts are reported in ppm, and the following abbreviations are used in reporting the NMR data: d (doublet), dd (doublet of doublets), m (multiplet). Coupling constants (*J*) are given in Hz. Spectra are reported as follows: chemical shift (δ, ppm), multiplicity, integration, coupling constants (Hz). Liquid chromatography (HPLC) was done using a Knauer apparatus equipped with a UV‑VIS detector. For monitoring of the reaction progress and postreaction mixtures, a LiChrospher 18-RP 10 μm column (4x240 mm) and 70 % methanol as the eluent at a flow rate of 1.3 cm^3^ min^-1^ were used. MS spectra were recorded on a Shimadzu LCMS IT-TOF spectrometer equipped with a Kinetex® 2.6 µm C18 100 Å, LC Column 100 x 2.1 mm, eluted with 50% aqueous acetonitrile at a flow rate of 0.3 cm^3^ min^-1^

Synthesis of 3-nitroprop-1-ene (**3**, C_3_H_5_NO_2_)

The powdered silver nitrite (50 g) was suspended in 100 cm^3^ of anhydrous diethyl ether at -5 ^o^C. Next, 35 g of 3-bromoprop-1-ene was slowly added dropwise while the temperature was maintained at -5 ^o^C. The mixture was then left at 0-5 °C for 24 hours, and next for 48 h at room temperature. The reaction mixture was filtered and the residue was washed with the diethyl ether. The filtrate and the ethereal extract were combined and the solvent was evaporated on a rotary evaporator. The liquid residue was distilled under reduced pressure. 7.2 g (29%) of 3-nitroprop-1-ene were obtained as a yellow liquid with a characteristic odor (bp. 35 ^o^C/10 mmHg; Ref.: 44 ^o^C/27 mmHg).

Synthesis of 5-nitromethyl-3-phenyl-4,5-dihydroisoxazole (**4**, C_10_H_10_N_2_O_3_)

A mixture of 40 mmol of 3-nitroprop-1-ene and 20 mmol of phenylhydraxamoyl chloride in 100 cm^3^ of dry Et_2_O was stirred and cooled down to 0 ^o^C. 20 mmol of Et_3_N in 10 cm^3^ of Et_2_O was added dropwise and the mixture was stirred at r.t. for 20 h. The organic layer was separated from the postreaction mixture, dissolved in DCM, washed with water, and dried over MgSO_4_. The solvent was evaporated to dryness and residues were crystallized from EtOH.

Yield 80%. White crystals. m.p.: 94-95 ˚C.

Anal. Calc. for C_10_H_10_N_2_O_3_ (206,20): C, 58.25; H, 4.89; N, 13.59; Found, %: C, 58.14; H, 4.78; N, 13.47.

HRMS (ESI): m/z = 207.0758 [C_10_H_10_N_2_O_3_ + H]^+^, m/z (theor.) = 207.0764, diff. = 2.90 ppm.

NMR experiments for **4**

^1^H NMR (500 MHz, CDCl_3_): 7,59 (m, 2H, Ar), 7,37 (m, 3H, Ar), 5,33 (dddd, 2H, *J =* 10,6Hz, 6,6Hz, 6,3Hz, 6,2Hz, H5), 4.62 (dd, 1H, *J* = 13.2, 6.6 Hz, H6a), 4.43 (dd, 1H, *J* = 13.2, 6.3 Hz, H6b), 3.58 (dd, *J* = 17.0, 10.6 Hz, 1H, H4a), 3.20 (dd, *J* = 17.0, 6.2 Hz, 1H, H4b). ^13^C NMR (125 MHz, CDCl_3_): 156,9 (C3), 130.8 (Ar_C4’_), 128,9 (Ar_C3’+C5’_), 128,4 (Ar_C1’_), 126,9 (Ar_C2’+C6’_), 76.8(C5) 76.1 (-CH_2_-), 38.7 (C4).

The ^1^H-NMR spectrum contains signals reflecting the ABMNX spin system, which belong to methylene protons and protons associated with the isoxazoline ring (Fig. S.1.1 A). Two geminal proton (H4 and H4') signals (as a doublet of doublets) are in a stronger field (δ = 3.20 ppm and δ = 3.58 ppm, respectively), whereas the last proton from the isoxazoline system (H5) is in a relatively weaker field (δ = 5.33 ppm). Next, the signals of protons from the nitro methyl group (Ha and Ha') are observed as two doublets of doublets in a moderate field (δ = 4.43 ppm, δ = 4.62 ppm). The number of resonance lines in respective multiplets confirmed that both protons of the exocyclic -CH_2_- moiety are not magnetically equivalent and are diastereotopic. Lastly, the multiplets corresponding to the five aryl protons are shifted in the evidently weakest field and appear at δ > 7 ppm. In the ^13^C-NMR spectrum (Fig. S.1.1.B), signals corresponding to phenyl and isoxazoline rings are observed in a very similar field as in the case of unsubstituted 3-phenyl-4,5-dihydroisoxazole. The signals of -CH_2_- carbon atoms were found at 38.64 (C4) and 76.80 (Ca) ppm. The position of the signals corresponding to the -CH_2_- carbon atoms allows a statement that the nitromethyl group is attached in position 5 of the isoxazole ring, and the internal -CH_2_- group is not connected with heteroatoms, since the signal of the -CH_2_- carbon atom attached to the oxygen should be shifted into a region of much higher frequencies (to about 75 ppm).

**Fig. S.1.1.** Key fragments of 1H-NMR and 13C spectra of dihydroisoxazole **4**

Experimental and calculated IR spectra of **4**

IR (KBr) 3438, 2927, 2359, 1601, 1545, 1497, 1446, 1412, 1387, 1360, 1224, 1177, 1077, 1012, 912, 893, 822, 769, 712, 692, 546, 483, 438 cm^-1^.

The NO_2_ stretch and bend frequencies are located at 1545.46 cm^-1^ and 1360.31 cm^-1^, respectively. However, DFT/6-31G(d) computation with the m06-2x functional yields 1781.93 cm^-1^ and 1523.92 cm^-1^. A better result is achieved with the b3lyp functional with the same basis set. It is 1680.85 cm^-1^ for the stretch and 1438.49 cm^-1^ for the bend frequencies.

*
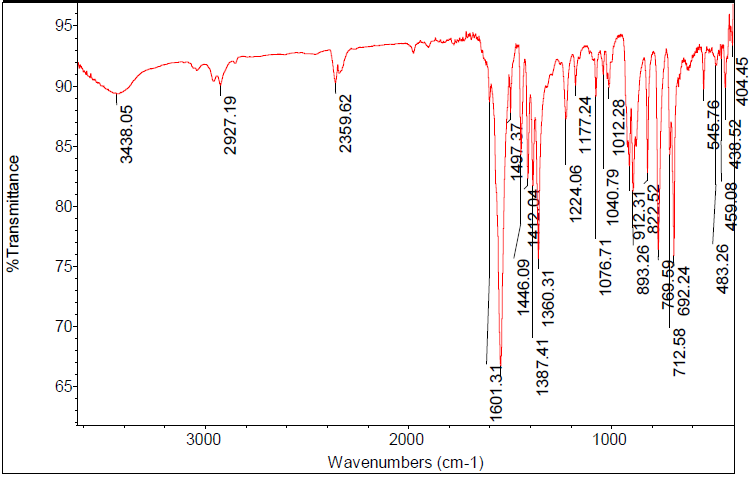
*

**Fig. S.1.2.** Experimental IR spectrum of 3-phenyl-5-nitromethyl-4,5-dihydroisoxazole

The calculated IR-spectrum of **4** (B3LYP/6-31G(d) theoretical level) is presented in Fig. S.1.3 and it is similar to the experimental one. Besides the above-mentioned NO_2_ vibrations, there are also the most prominent N-O peaks within the isoxazole ring at 952.75 cm^-1^, CH_2_-bridge at 1397.10 cm^-1^, and CH-stretch in the phenyl ring at 3203.53 cm^-1^.


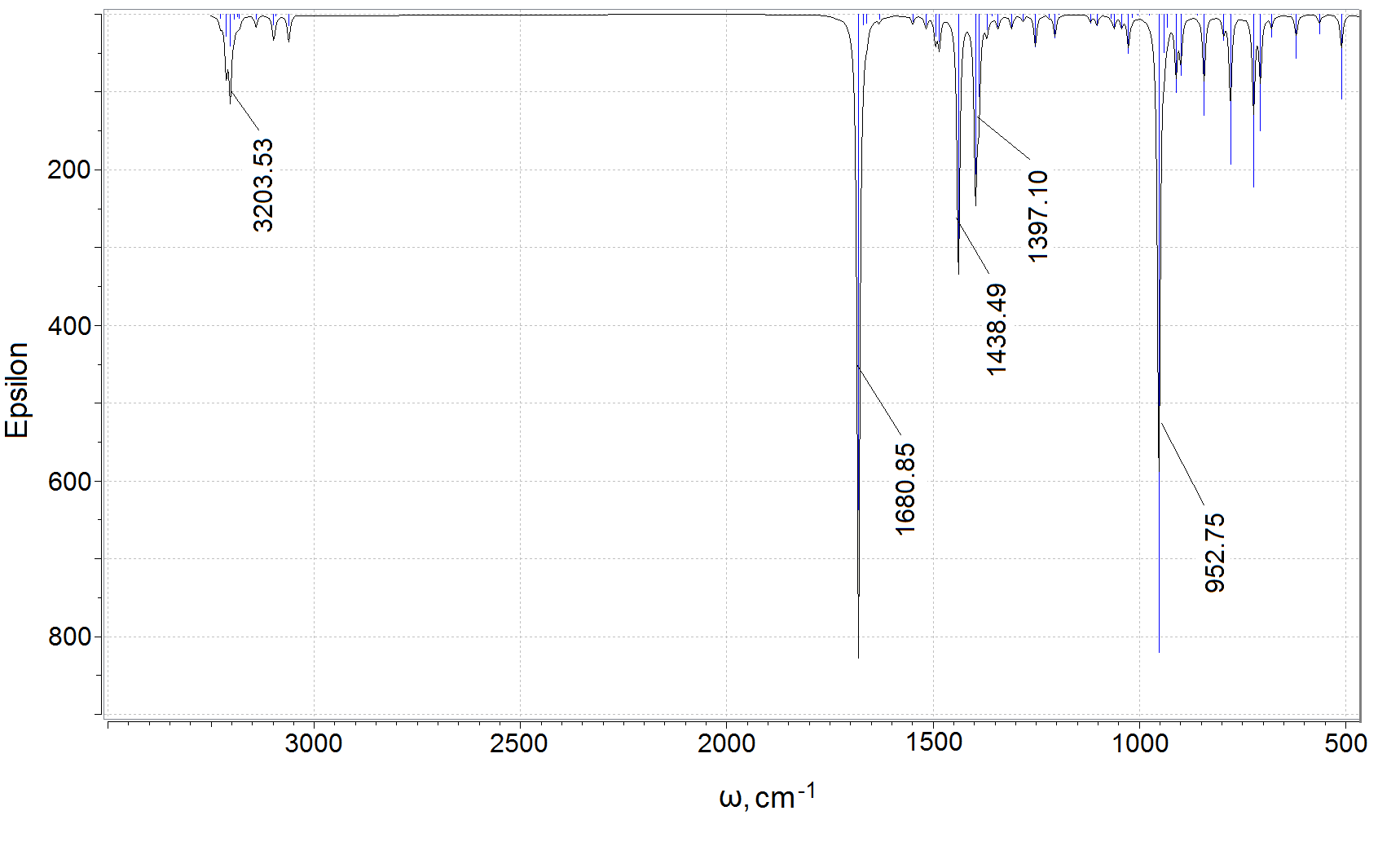


**Fig. S.1.3.** Calculated IR spectrum of **4**

Experimental and calculated UV-VIS spectra of **4**

To model the UV-VIS spectrum, TDDFT/6-31G(d) calculation was carried out for 10 excited singlet states using PCM with methanol as a solvent. Here, B3LYP performs better than M062X as well. The maximum of the widest band in the experimental spectrum (Fig. S.1.4) is located at 259 nm.

*
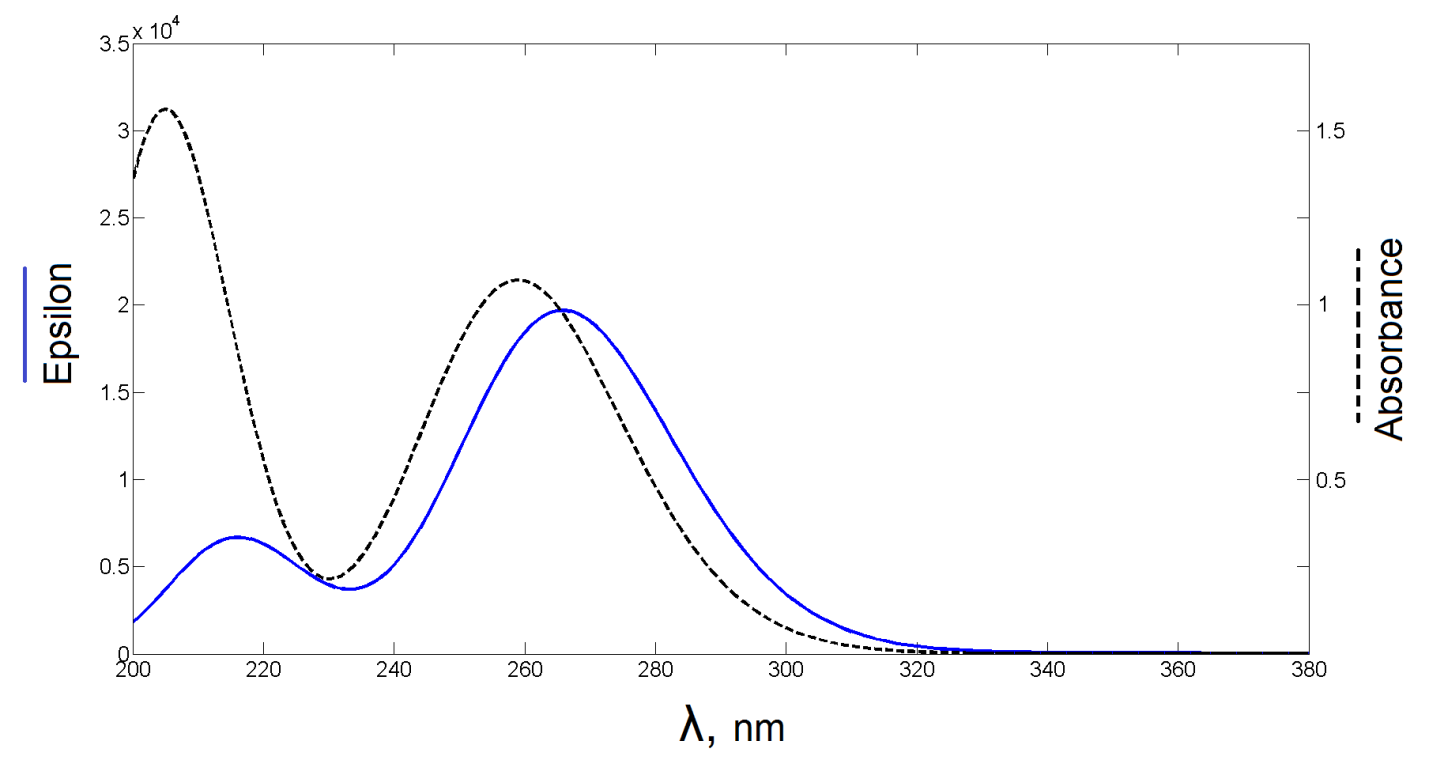
*

**Fig. S.1.4.**Modeled, based on TDDFT calculation (solid), and experimental (dotted) UV spectrum of 5-nitromethyl-3-phenyl-4,5-dihydroisoxazole taken in methanol

The HOMO→LUMO transition is very weak with an oscillator strength value of 0.0012. It is barely seen in Fig. 8 at 350.23 nm. The experimental spectrum reveals the peak at 358 nm with an absorbance value of 3.4761*10^-4^ AU. The strongest transition is found between HOMO and LUMO+1 with oscillator strength f=0.4772. The corresponding wavelength is 265.97 nm. The plots of these MOs are shown in Fig. 9. The NO_2_ group has no electron density within HOMO and LUMO+1. Similarly, the phenyl and isoxazole rings are empty within LUMO.

*
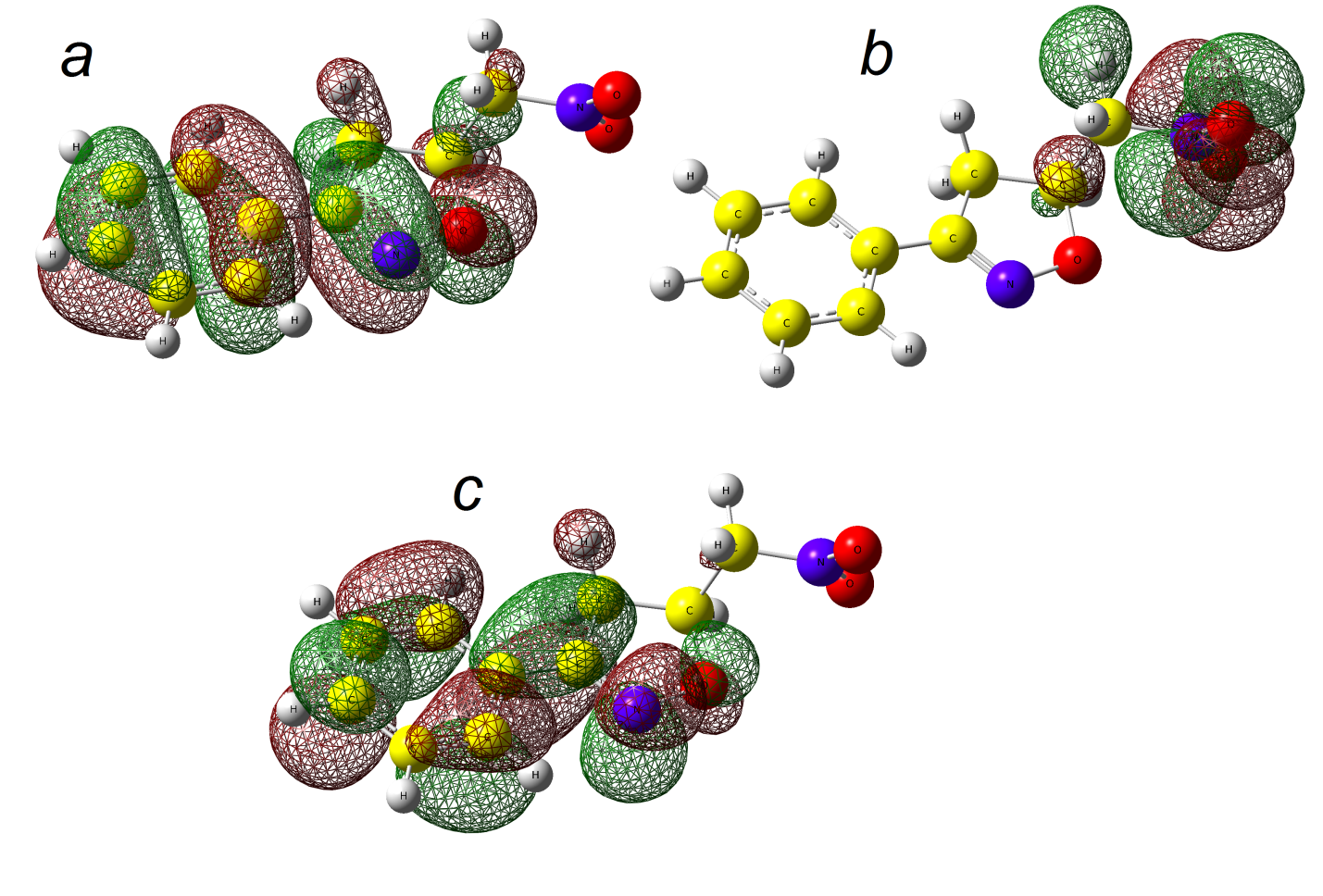
*

**Fig. S.1.5.** Molecular orbitals of **4**: a – HOMO; b – LUMO; c – LUMO+1

The left peak in Fig. S.1.5. located at 215.61 nm is due to the excited state consisting of 6 transitions. The largest contributions come from HOMO→LUMO+2 and HOMO-1→LUMO+1. In the experimental spectrum, this peak is located at 205 nm and is higher than the calculated one. However, within this level of computation, the results are quite satisfactory.

*X-Ray Crystallography*

Crystals of **4** were obtained by recrystallization from ethanol. The crystals were twinned; however, the diffraction data produced good quality electron density maps that allowed us to unambiguously determine the structural model of the crystal (Table S.1.1.). The diffraction data were collected at room temperature on a XtaLAB AFC11 (RCD3): quarter-chi single diffractometer using CuK*α* radiation (*λ* = 1.54184 Å). Using Olex2 [44], the structure was solved with the ShelXT [45] structure solution program using Intrinsic Phasing and refined with the Olex2.refine refinement package using Gauss-Newton minimisation.

**Table S.1.1.** Crystal data and structure refinement parameters for 5-nitromethyl-3-phenyl-4,5-dihydroisoxazole **4**

| Formula | C_10_H_10_N_2_O_3_ |
| --- | --- |
| Formula weight | 206.20 |
| Temperature/K | 295 |
| Crystal system | orthorhombic |
| Space group | *Pbca* |
| a/Å | 8.5754(11) |
| b/Å | 9.8973(9) |
| c/Å | 23.684(3) |
| Volume/Å^3^ | 2010.2(4) |
| Z | 8 |
| ρ_calc_g/cm^3^ | 1.3626 |
| μ/mm^‑1^ | 0.861 |
| F(000) | 867.1 |
| Crystal size/mm^3^ | 0.01 × 0.2 × 0.2 |
| Radiation | Cu K*α* (*λ* = 1.54184) |
| 2Θ range for data collection/° | 7.46 to 145.58 |
| Reflections collected | 28948 |
| Independent reflections | 1853 [*R_int_* = 0.3519, *R_sigma_* = 0.0552] |
| Data/parameters | 1853/135 |
| Goodness-of-fit on F^2^ | 0.817 |
| Final R indexes [I>=2σ (I)] | *R*_1_ = 0.0935, *wR*_2_ = 0.2952 |
| Largest diff. peak/hole / e Å^-3^ | 0.36/-0.24 |
| CCDC No. | 1576601 |

CCDC 1576601 contains supplementary crystallographic data for this paper. These data can be obtained free of charge via <http://www.ccdc.cam.ac.uk/conts/retrieving.html> (or from the Cambridge Crystallographic Data Centre, 12, Union Road, Cambridge CB2 1EZ, UK; fax: +44 1223 336033).

**
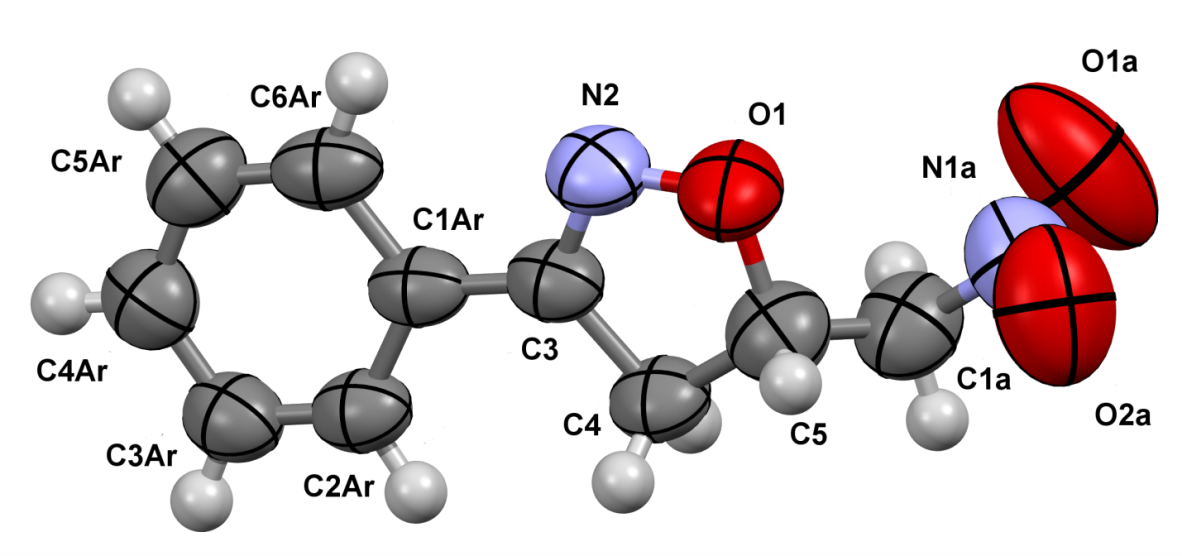
**

**Fig. S.1.6.** Molecular structure of **4**


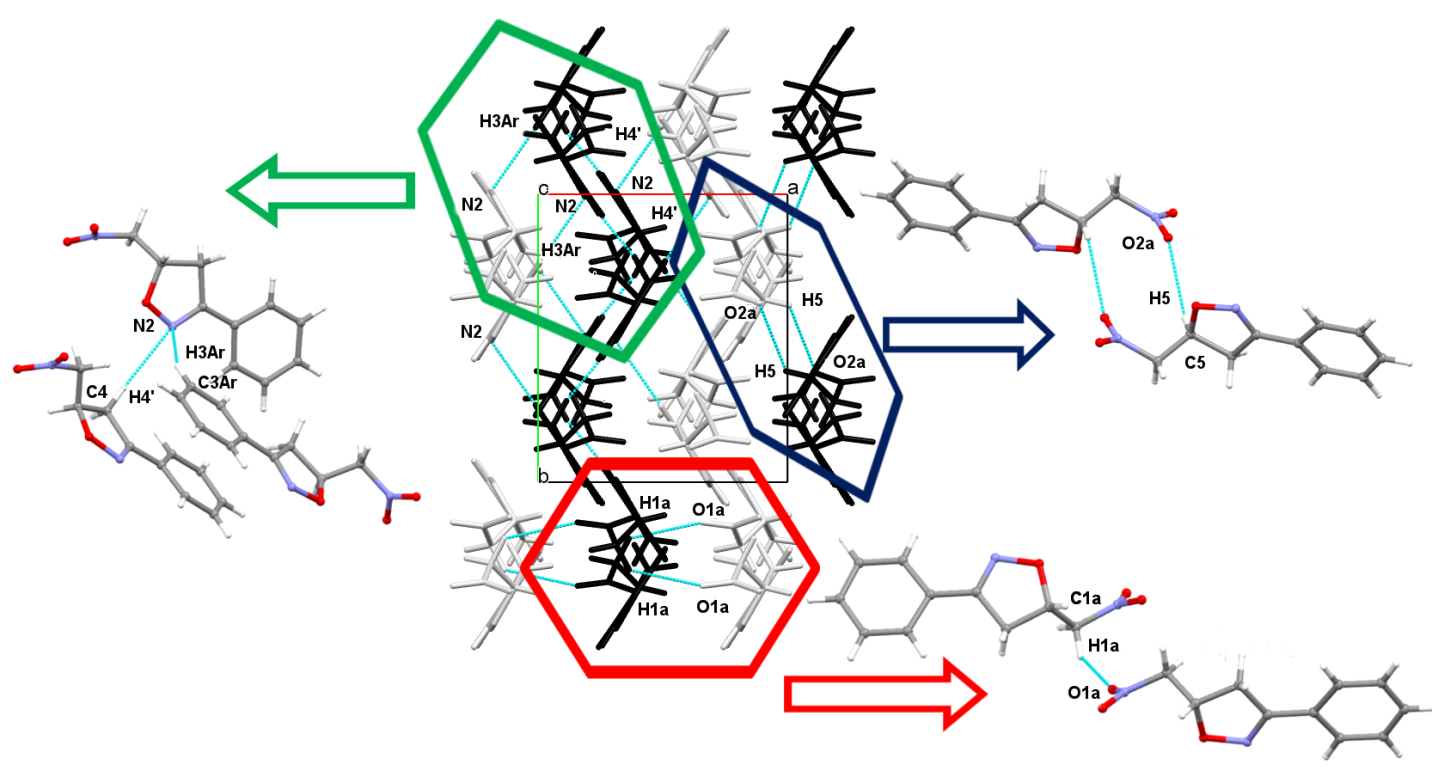


**Fig. S.1.7.** Crystal packing (view along the *c* axis) and selected intermolecular interactions in **4**. Two directions of columns of molecules oriented in a head-to-tail manner are marked in grey and black

ECP and charge distribution of **4**

Similarly useful information in prediction of the properties of the studied substance is the charge distribution within the molecule as well the electrostatic potential (ESP) and bond order. The charge distribution was calculated to fit the ECP according to the Merz-Singh-Kollman (MK) scheme. Fig. S.1.8. presents the ECP surface and local charges on atoms.


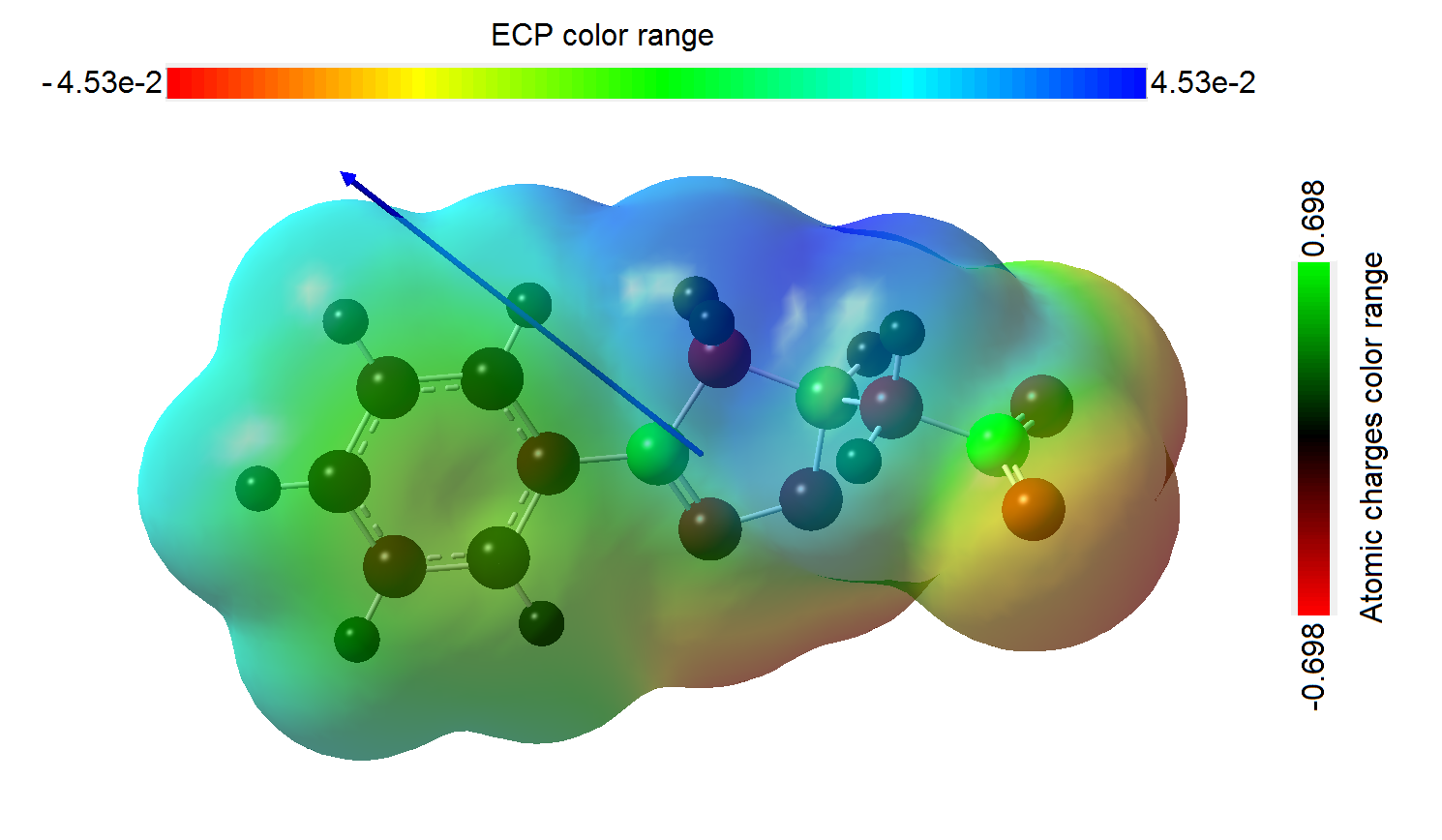


**Fig. S.1.8.** ECP and charge distribution over a molecule of **4**

The blue arrow shows the direction of the dipole moment. Its magnitude is 6.313D. The molecule has two main negative sites concentrated around the –NO_2_ group and =N-O- within the isoxazole ring. The positive site extends above the opposite part of the ring and the external -CH_2_- group. The space around the phenyl ring is relatively neutral.

Natural bond analysis for **4**

Finally, natural bond analysis for the molecule was carried out and bond indices were computed. The bond orders for all neighbour atoms except hydrogen are plotted in Fig. S.1.9. Two –N=O bonds within the nitro group are not identical due to steric factors. This is also seen in Fig. 10 where two oxygen atoms have a slightly different charge value. The order of the C3=N2 bond in the isoxazole ring is only 1.772. This is caused by partial conjugation with the aromatic phenyl ring. Also the C3-C1Ar bond is denser than an ordinary single bond. At the same time, the aromatic bonds between C1Ar-C2Ar and C1Ar-C6Ar are weaker due to the conjugation.


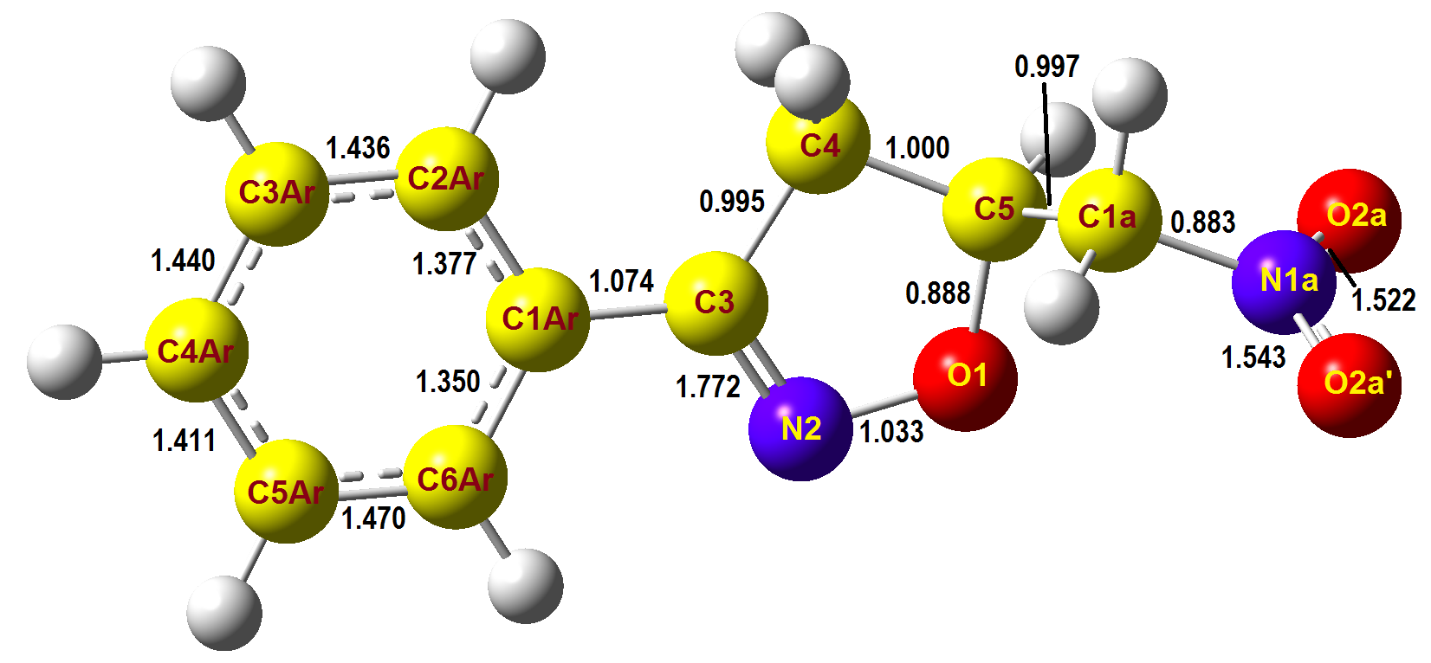


**Fig. S.1.9.** Bond orders between neighbor atoms of molecule (**4**)

**[2] Theoretical study of [3+2] cycloaddition between benzonitrile N-oxides and 3-nitroprop-1-ene**

**Table S.2.1.** Energetic parameters for [3+2] cycloadditions leading to 5-nitromethyl-3-phenyl-4,5-dihydroisoxazole (**4**) and regioisomeric 4-nitromethyl-3-phenyl-4,5-dihydroisoxazole (**5**) according to M06-2X/6-31G(d) (PCM) calculations

| Solvent | Transition | ΔH [kJ/mol] | ΔG [kJ/mol] | ΔS  [J/mol∙K] |
| --- | --- | --- | --- | --- |
| Diethyl | **2**+**3**→**MC** | -27.2 | 19.3 | -156.2 |
| Ether | **MC**→**TSA** | 76.2 | 87.1 | -192.8 |
| (ε=4.34) | **2**+**3**→**4** | -213.0 | -151.0 | -208.0 |
|  | **MC**→**TSB** | 84.6 | 96.1 | -194.7 |
|  | **2**+**3**→**5** | -201.3 | -139.0 | -208.6 |
| Dichloromethane | **2**+**3**→**MC** | -24.5 | 19.3 | -146.7 |
| (ε=8.93) | **MC**→**TSA** | 73.6 | 86.6 | -190.5 |
|  | **2**+**3**→**4** | -213.8 | -152.2 | -206.5 |
|  | **MC**→**TSB** | 83.8 | 96.5 | -189.1 |
|  | **2**+**3**→**5** | -200.1 | -138.8 | -205.7 |
| Acetone | **2**+**3**→**MC** | -23.4 | 20.5 | -147.3 |
| (ε=20.70) | **MC**→**TSA** | 72.8 | 86.0 | -191.4 |
|  | **2**+**3**→**4** | -214.1 | -152.0 | -208.3 |
|  | **MC**→**TSB** | 84.4 | 96.3 | -187.1 |
|  | **2**+**3**→**5** | -199.2 | -137.7 | -206.3 |
| Nitromethane | **2**+**3**→**MC** | -22.9 | 21.5 | -149.0 |
| (ε=38.20) | **MC**→**TSA** | 72.5 | 85.7 | -193.4 |
|  | **2**+**3**→**4** | -214.1 | -151.3 | -210.9 |
|  | **MC**→**TSB** | 84.5 | 96.7 | -189.7 |
|  | **2**+**3**→**5** | -198.8 | -136.6 | -208.7 |
| Water | **2**+**3**→**MC** | -22.6 | 24.6 | -158.3 |
| (ε=78.39) | **MC**→**TSA** | 72.3 | 85.6 | -203.0 |
|  | **2**+**3**→**4** | -214.1 | -148.3 | -220.9 |
|  | **MC**→**TSB** | 84.6 | 97.0 | -199.7 |
|  | **2**+**3**→**5** | -198.5 | -133.5 | -218.6 |

**Table S.2.2.** Most important parameters for the key structures of [3+2] cycloadditions leading to 5-nitromethyl-3-phenyl-4,5-dihydroisoxazole (**4**) and regioisomeric 4-nitromethyl-3-phenyl-4,5-dihydroisoxazole (**5**) according to M06-2X/6-31G(d) (PCM) calculations

|  | Structure | C3-C4 | | C5-O1 | | GEDT  [e] | Imaginary  frequencies  [cm^-1^] |
| --- | --- | --- | --- | --- | --- | --- | --- |
|  |  | r [Å] | l^a^ | r [Å] | l^a^ |  |  |
| Diethyl Ether  (ε=4.34) | **TSA** | 2.192 | 0.549 | 2.281 | 0.416 | 0.002 | -462.63 |
|  | **4** | 1.511 |  | 1.440 |  |  |  |
|  | **TSB** | 2.213 | 0.540 | 2.221 | 0.457 | 0.005 | -475.79 |
|  | **5** | 1.516 |  | 1.439 |  |  |  |
| Dichloromethane  (ε=8.93) | **TSA** | 2.188 | 0.552 | 2.288 | 0.412 | 0.007 | -465.80 |
|  | **4** | 1.511 |  | 1.441 |  |  |  |
|  | **TSB** | 2.212 | 0.541 | 2.223 | 0.455 | 0.005 | -478.66 |
|  | **5** | 1.516 |  | 1.439 |  |  |  |
| Acetone  (ε=20.70) | **TSA** | 2.186 | 0.553 | 2.292 | 0.409 | 0.010 | -467.71 |
|  | **4** | 1.511 |  | 1.441 |  |  |  |
|  | **TSB** | 2.215 | 0.539 | 2.222 | 0.457 | 0.003 | -478.70 |
|  | **5** | 1.516 |  | 1.440 |  |  |  |
| Nitromethane (ε=38.20) | **TSA** | 2.186 | 0.553 | 2.294 | 0.409 | 0.011 | -468.31 |
|  | **4** | 1.511 |  | 1.442 |  |  |  |
|  | **TSB** | 2.215 | 0.540 | 2.223 | 0.456 | 0.002 | -479.25 |
|  | **5** | 1.517 |  | 1.440 |  |  |  |
| Water (ε=78.39) | **TSA** | 2.185 | 0.556 | 2.300 | 0.405 | 0.012 | -468.93 |
|  | **4** | 1.511 |  | 1.442 |  |  |  |
|  | **TSB** | 2.216 | 0.539 | 2.223 | 0.456 | 0.002 | -479.66 |
|  | **5** | 1.517 |  | 1.440 |  |  |  |

^a^$l_{X-Y}=1-\frac{r_{X-Y}^{TS}-r_{X-Y}^{P}}{r_{X-Y}^{P}}$

where $r_{X-Y}^{TS}$ is the distance between the reaction centres X and Y in the transition structure and $r_{X-Y}^{P}$ is the same distance in the corresponding product.

Cartesian coordinates of key structures of [3+2] cycloadditions leading to 5-nitromethyl-3-phenyl-4,5-dihydroisoxazole (**4**) and regioisomeric 4-nitromethyl-3-phenyl-4,5-dihydroisoxazole (**5**) in diethyl ether according to M06-2X/6-31G(d) (PCM) calculations

**2**

---------------------------------------------------------------------

Center Atomic Atomic Coordinates (Angstroms)

Number Number Type X Y Z

---------------------------------------------------------------------

1 7 0 -0.433982 0.820532 -0.629159

2 6 0 0.708952 0.730643 -0.465500

3 6 0 2.127067 0.691522 -0.247244

4 6 0 2.846843 1.890368 -0.156344

5 6 0 2.782814 -0.539616 -0.119842

6 6 0 4.217968 1.849687 0.064715

7 1 0 2.328481 2.837779 -0.258672

8 6 0 4.153947 -0.565454 0.104593

9 1 0 2.217042 -1.461630 -0.200497

10 6 0 4.871815 0.625706 0.197679

11 1 0 4.776629 2.777238 0.134319

12 1 0 4.662310 -1.518693 0.205747

13 1 0 5.942526 0.600295 0.372709

14 8 0 -1.637447 0.886658 -0.798839

---------------------------------------------------------------------

**3**

---------------------------------------------------------------------

Center Atomic Atomic Coordinates (Angstroms)

Number Number Type X Y Z

---------------------------------------------------------------------

1 6 0 -0.586781 -2.346252 -0.117641

2 1 0 0.006765 -2.125713 0.767224

3 1 0 -0.089816 -2.862700 -0.932636

4 6 0 -1.871282 -2.012254 -0.191732

5 1 0 -2.469449 -2.240964 -1.068414

6 6 0 -2.586539 -1.369540 0.954031

7 1 0 -3.163958 -2.101202 1.531588

8 1 0 -1.923182 -0.828691 1.628250

9 7 0 -3.626151 -0.376615 0.519060

10 8 0 -4.220331 -0.590005 -0.518534

11 8 0 -3.851768 0.545270 1.277934

---------------------------------------------------------------------

**MC**

---------------------------------------------------------------------

Center Atomic Atomic Coordinates (Angstroms)

Number Number Type X Y Z

---------------------------------------------------------------------

1 7 0 0.301449 -0.945160 -0.629852

2 6 0 -0.798300 -0.641028 -0.431767

3 6 0 -2.175206 -0.333265 -0.167907

4 6 0 -3.096292 -1.374789 0.006434

5 6 0 -2.590417 1.001568 -0.079549

6 6 0 -4.426685 -1.074001 0.271208

7 1 0 -2.763337 -2.404674 -0.066339

8 6 0 -3.923364 1.287914 0.189390

9 1 0 -1.870556 1.799968 -0.224879

10 6 0 -4.841104 0.253675 0.365401

11 1 0 -5.141014 -1.879660 0.405286

12 1 0 -4.245491 2.321628 0.260432

13 1 0 -5.881041 0.482455 0.574905

14 8 0 1.464789 -1.237318 -0.836760

15 6 0 1.053832 2.149481 -0.235388

16 1 0 0.463340 2.071113 0.675160

17 1 0 0.630554 2.723240 -1.053646

18 6 0 2.250932 1.580514 -0.336451

19 1 0 2.847777 1.666307 -1.239279

20 6 0 2.877679 0.852653 0.810220

21 1 0 3.601431 1.482197 1.341417

22 1 0 2.151621 0.466024 1.524705

23 7 0 3.699146 -0.329351 0.381323

24 8 0 4.283222 -0.262948 -0.681519

25 8 0 3.779002 -1.252652 1.167328

---------------------------------------------------------------------

**TSA**

---------------------------------------------------------------------

Center Atomic Atomic Coordinates (Angstroms)

Number Number Type X Y Z

---------------------------------------------------------------------

1 7 0 -0.455407 0.926419 -0.694807

2 6 0 0.547739 0.316741 -0.446804

3 6 0 1.972373 0.464019 -0.239526

4 6 0 2.538036 1.748254 -0.222607

5 6 0 2.783302 -0.659888 -0.056713

6 6 0 3.904519 1.894622 -0.026713

7 1 0 1.900952 2.614944 -0.365023

8 6 0 4.150876 -0.499953 0.141214

9 1 0 2.347715 -1.652873 -0.070552

10 6 0 4.713828 0.773314 0.156613

11 1 0 4.338795 2.889118 -0.016236

12 1 0 4.776215 -1.375186 0.283372

13 1 0 5.781112 0.894187 0.311277

14 8 0 -1.667897 0.795226 -0.835845

15 6 0 -0.345145 -1.670613 -0.203812

16 1 0 0.083391 -1.766346 0.792036

17 1 0 0.216556 -2.115074 -1.019936

18 6 0 -1.681243 -1.435054 -0.356911

19 1 0 -2.180506 -1.612157 -1.302777

20 6 0 -2.561042 -1.222420 0.834461

21 1 0 -3.054601 -2.149575 1.147849

22 1 0 -2.029839 -0.786764 1.679750

23 7 0 -3.697897 -0.290291 0.531176

24 8 0 -4.301681 -0.466664 -0.508825

25 8 0 -3.968355 0.551998 1.363772

---------------------------------------------------------------------

**4**

---------------------------------------------------------------------

Center Atomic Atomic Coordinates (Angstroms)

Number Number Type X Y Z

---------------------------------------------------------------------

1 7 0 0.148881 -0.993830 -0.811280

2 6 0 -0.507804 0.045408 -0.456432

3 6 0 -1.939570 -0.019796 -0.126262

4 6 0 -2.607568 -1.251366 -0.096321

5 6 0 -2.646920 1.152226 0.155303

6 6 0 -3.960484 -1.302849 0.208106

7 1 0 -2.053779 -2.158922 -0.313245

8 6 0 -4.005212 1.096337 0.458780

9 1 0 -2.140629 2.113143 0.132854

10 6 0 -4.663400 -0.129110 0.486550

11 1 0 -4.470972 -2.260501 0.230988

12 1 0 -4.546524 2.012049 0.674122

13 1 0 -5.721391 -0.172545 0.725487

14 8 0 1.465803 -0.650945 -1.092168

15 6 0 0.325353 1.306495 -0.461981

16 1 0 0.223344 1.880680 0.463575

17 1 0 0.041993 1.953128 -1.298439

18 6 0 1.713688 0.691334 -0.633883

19 1 0 2.333093 1.182873 -1.383508

20 6 0 2.447722 0.601493 0.691722

21 1 0 2.714495 1.589573 1.077123

22 1 0 1.882173 0.046725 1.441238

23 7 0 3.746424 -0.130983 0.528348

24 8 0 4.372839 0.057232 -0.496896

25 8 0 4.103389 -0.837980 1.448669

---------------------------------------------------------------------

**TSB**

---------------------------------------------------------------------

Center Atomic Atomic Coordinates (Angstroms)

Number Number Type X Y Z

---------------------------------------------------------------------

1 8 0 -1.136943 3.055459 -0.199278

2 7 0 -0.083663 2.442289 -0.052527

3 6 0 0.390194 1.341913 -0.127582

4 6 0 1.605355 0.578477 0.061396

5 6 0 2.696312 1.177380 0.710083

6 6 0 1.698903 -0.742727 -0.391710

7 6 0 3.869201 0.457757 0.890750

8 1 0 2.612382 2.200485 1.061286

9 6 0 2.878912 -1.454230 -0.198684

10 1 0 0.855480 -1.219872 -0.882437

11 6 0 3.963934 -0.858449 0.438302

12 1 0 4.711867 0.926402 1.388753

13 1 0 2.946946 -2.478060 -0.551094

14 1 0 4.882218 -1.417735 0.585179

15 6 0 -2.337861 1.312368 -0.871969

16 6 0 -1.461110 0.275948 -0.703892

17 1 0 -2.445815 1.798659 -1.833893

18 1 0 -0.949068 -0.137341 -1.569159

19 1 0 -3.084474 1.543670 -0.119522

20 6 0 -1.579214 -0.631223 0.493899

21 1 0 -0.622145 -0.891061 0.954665

22 1 0 -2.262473 -0.240247 1.246195

23 7 0 -2.139065 -1.958754 0.058255

24 8 0 -3.148783 -2.365394 0.594989

25 8 0 -1.523606 -2.548728 -0.813544

---------------------------------------------------------------------

**5**

---------------------------------------------------------------------

Center Atomic Atomic Coordinates (Angstroms)

Number Number Type X Y Z

---------------------------------------------------------------------

1 8 0 -1.206134 2.564081 -0.035577

2 7 0 0.115526 2.179476 0.102027

3 6 0 0.245506 0.937540 -0.182533

4 6 0 1.537259 0.240812 -0.087003

5 6 0 2.667717 0.907612 0.404963

6 6 0 1.647983 -1.095676 -0.483582

7 6 0 3.883330 0.244825 0.495448

8 1 0 2.578305 1.944279 0.712813

9 6 0 2.869436 -1.757952 -0.387434

10 1 0 0.783671 -1.625697 -0.875212

11 6 0 3.987877 -1.090492 0.101336

12 1 0 4.754177 0.768669 0.876823

13 1 0 2.943563 -2.795163 -0.697974

14 1 0 4.940111 -1.606104 0.176062

15 6 0 -1.905036 1.538294 -0.763340

16 6 0 -1.079299 0.280431 -0.517311

17 1 0 -1.913074 1.803559 -1.825488

18 1 0 -1.029630 -0.368746 -1.394717

19 1 0 -2.926849 1.491242 -0.380335

20 6 0 -1.554018 -0.529212 0.695132

21 1 0 -0.844918 -1.312369 0.967670

22 1 0 -1.782309 0.110790 1.547147

23 7 0 -2.821948 -1.226882 0.321294

24 8 0 -2.726094 -2.143488 -0.473908

25 8 0 -3.863100 -0.812557 0.793973

---------------------------------------------------------------------
